# The ‘see-and-treat’ approach in the treatment of preneoplastic cervical lesions

**DOI:** 10.1097/CEJ.0000000000001002

**Published:** 2026-02-25

**Authors:** Ermelinda Monti, Camilla Monti, Cristina Maria Michela Matozzo, Marta Salmaso, Maria Pasquali Coluzzi, Eugenia Di Loreto, Giada Libutti, Veronica Boero, Daniela Alberico, Giussy Barbara

**Affiliations:** a Gynecology Unit, Fondazione IRCCS Ca’ Granda Ospedale Maggiore Policlinico; b Department of Clinical Sciences and Community Health, Università degli Studi di Milano; c Obstetric and Gynecological Emergency Unit and Service for Sexual and Domestic Violence, SVSeD, Fondazione IRCCS Ca’ Granda Ospedale Maggiore Policlinico, Milan, Italy

## Abstract

This study evaluated the overtreatment rate (defined as a histological finding of low-grade intraepithelial lesion or negative after cervical excisional procedures) associated with the ‘see-and-treat’ approach in patients undergoing cervical conization for citology-detected suspected high-grade cervical lesions. The ‘see-and-treat’ strategy consists of colposcopy and immediate excision without prior cervical biopsy when a high-grade lesion is suspected based on colposcopic impression. Secondary objectives were to compare this cohort with patients treated after histological confirmation of high-grade lesions and to identify clinical and epidemiological factors associated with increased overtreatment risk, to better select candidates for ‘see-and-treat’. We conducted a retrospective monocentric cohort study on women over 25 years of age with high-grade cytology who underwent an excisional procedure with or without a previous cervical biopsy. Overtreatment was defined as histopathology showing cervical intraepithelial neoplasia grade 1 or negative findings. Of the 892 patients included, 643 underwent the ‘see-and-treat’ approach. The overall overtreatment rate in this group was 13.5%, and 6% among women with both high-grade cytology and high-grade colposcopic impression (high-concordance subgroup). The factors most strongly associated with overtreatment were low-grade or negative colposcopic impression [odds ratio (OR): 7.36] and nonvisible squamocolumnar junction (OR: 3.57). Menopause (OR: 4.5, *P* < 0.0001) and atypical squamous cells – cannot exclude HSIL cytology (OR: 2.97, *P* < 0.0001) also increased the risk, whereas human papillomavirus 16/18 positivity was associated with a lower risk (*P* = 0.004). In the high-concordance subgroup, none of these factors showed significant correlation with overtreatment. The ‘see-and-treat’ approach appears particularly advantageous in cases with high-grade concordance, offering a low overtreatment rate and allowing the detection of 2.8% of invasive cancers that might have been missed by biopsy. Moreover, it significantly reduces the interval between colposcopy and treatment (29 vs. 68 days). These findings support the selective use of ‘see-and-treat’ in appropriately stratified patients.

## Introduction

The ‘see-and-treat’ approach is defined as a treatment strategy that involves performing colposcopy and the excisional procedure without a prior cervical biopsy, in cases where a high-grade lesion is suspected based on colposcopic impression. Combining the diagnostic and therapeutic procedure without the requirement for an intermediate biopsy may reduce costs, the time needed for histological diagnosis, the number of cervical cancers missed in follow-up, and the potential increase in patient anxiety and worry over time, thereby enhancing compliance (Ciavattini *et al*., 2019).

Excisional treatment may not necessarily be performed at the time of first colposcopy, but can be scheduled within a short time frame, provided that an appropriate informed consent is obtained.

The ‘two-step’ procedure consists of an initial colposcopy with biopsy, followed by a subsequent treatment at a second visit if the biopsy results indicate cervical intraepithelial neoplasia 2 (CIN2) or greater (CIN2+). The overtreatment rate – defined as a histological finding of low-grade squamous intraepithelial lesions (LSILs) (CIN1) or negative results following cervical excisional procedures – for this procedure ranges between 11 and 35% (Ebisch *et al*., 2016).

The rationale behind a ‘see-and-treat’ protocol is that for women with a clinically significant risk of progression to cancer [i.e. suspected high-grade squamous intraepithelial lesion (HSIL) both cytologically and at colposcopic examination], or when there is an obvious need for treatment, there is little benefit in using a two-step approach that involves performing a biopsy and asking the patient to return for potential treatment once the histological result is available (Prendiville and Sankaranarayanan, 2017). An additional clinical advantage is that direct excision might offer greater diagnostic guarantees compared with the traditional ‘punch biopsy’. It can avoid the erroneous treatment of microinvasive carcinoma areas away from the biopsied area with destructive therapy (Diathermocoagulation, laser).

In contrast, this procedure presents a risk of overtreatment, which varies between 13 and 83%(Cárdenas-Turanzas *et al*, 2005; Ebisch *et al*., 2016). Based on literature data, this approach may be preferable in women over the age of 25, who are not pregnant and who have high-grade cytology, identifying an acceptable overtreatment rate of 10% of cases (Cárdenas-Turanzas *et al*, 2005; Ciavattini *et al*., 2019).

Many studies have been conducted on the ‘see-and-treat’ strategy, including different patient groups, a wide age range, and a variety of inclusion criteria for accessing this treatment modality (Kietpeerakool *et al*., 2007; Aue-Aungkul *et al*., 2011; Bosgraaf *et al*, 2013; Ebisch *et al*., 2016; Kuroki *et al*., 2016; Ciavattini *et al*., 2019; SICPCV Gestione delle lesioni istologiche, 2019; Loopik *et al*., 2020; Kiviharju *et al*., 2022; Rongthongaram *et al*., 2023). The findings of these studies show variable percentages of overtreatment for suspected high-grade intraepithelial lesions, which renders it challenging to ascertain in which cases the ‘see-and-treat’ management should be justified or should be avoided.

Based on this background, the primary aim of the present research was to investigate the risk of overtreatment in women undergoing cervical conization using the loop electrosurgical excision procedure (LEEP) with a ‘see-and-treat’ approach.

The secondary aim of this study was to make a comparison between the characteristics of patients treated with the ‘see-and-treat’ approach with those of a group of women who underwent the procedure after histological confirmation of high-grade lesions through biopsy (i.e. the ‘two-step’ procedure). Finally, we aimed to identify the main clinical and epidemiological factors associated with a higher risk of overtreatment in the studied population, to accurately select patients eligible for the ‘see-and-treat’ procedure.

## Materials and methods

A retrospective monocentric study was conducted on a group of patients undergoing cervical conization using LEEP between January 2013 and December 2023, following the detection of cytological high-grade intraepithelial abnormalities or suspected carcinoma. Patients were selected from the cohort attending the Regional Referral ‘Centre for Prevention, Diagnosis and Treatment of HPV-related Genital Disorders’, at the Gynecology Unit of the Fondazione IRCCS Ca’ Granda Ospedale Maggiore Policlinico, Milan, Italy.

The research project was approved by the local ethics committee (Ethics Committee of Milan area 2, protocol number #156025, 14 April 2022).

All women aged 25 or older, with a Pap smear suggestive of high-grade intraepithelial lesions and who underwent an excisional procedure with or without a previous cervical biopsy, were considered eligible. Exclusion criteria included age under 25 years, pregnancy at the time of treatment, low-grade cytological abnormalities, atypical squamous cells of undetermined significance or LSIL, and glandular cytological abnormalities atypical glandular cells (AGCs). The latter cytological results were included when concurrent with high-grade squamous lesions.

The patients’ demographic and medical history data, as well as the results of the cytology and colposcopy tests, were extracted from electronic outpatient clinical records. The histological outcomes of any biopsies and definitive treatment were provided by the Pathology Unit of the Fondazione IRCCS Ca’ Granda Ospedale Maggiore Policlinico.

Cytological outcomes were defined using the most recent Bethesda reporting system (2014) (Nayar and Wilbur, 2015).

High-grade cytological abnormalities were defined by the occurrence of the following cytological results: atypical squamous cells – cannot exclude HSIL (ASC-H), HSIL, or suspected squamous carcinoma or adenocarcinoma.

Colposcopic findings were reported in accordance with the 2011 International Federation for Cervical Pathology and Colposcopy guidelines (Bornstein *et al*., 2012).

The colposcopic patterns observed before proceeding with excisional treatment were defined as follows: (a) normal colposcopy or G0; (b) minor abnormal colposcopic findings or G1 characterized by ‘thin acetowhite epithelium’, ‘regular mosaic’, ‘regular punctation’, and (c) and major colposcopic findings or G2 if ‘dense acetowhite epithelium’, ‘irregular mosaic’, ‘irregular punctation’, ‘thickened glandular openings’, or ‘rapid onset of acetowhite reaction’ were present.

Information regarding the type of squamocolumnar junction (SCJ) at the time of colposcopy was also collected: visible exocervical SCJ (type 1), visible endocervical SCJ (type 2), and nonvisible SCJ (type 3).

The choice between the ‘see-and-treat’ approach and the conventional ‘two-step’ approach was made taking into consideration a number of factors, as for instance the Pap smear result, the colposcopic impression, the patients’ clinical and demographic history, and the patient preference. The excisional treatment using LEEP was performed on an outpatient basis under local anesthesia.

The definitive histological diagnosis, following the excision procedure, were as follows: negative, CIN1 (mild CIN), CIN2 (moderate CIN), CIN3 (severe CIN), adenocarcinoma in-situ (AIS), microinvasive carcinoma, or invasive carcinoma. The status of the histological sample margins (positive endocervical or exocervical, negative, or nonassessable) was also reported. The time elapsed between colposcopy and excisional treatment was calculated. Overtreatment was defined as the presence of a low-grade (CIN1) or negative posttreatment histological outcome.

Finally, the results of the ‘see-and-treat’ strategy were compared with those obtained in the population undergoing the conventional ‘two-step’ approach. In the two studied populations, the recurrence of cervical disease at 3 years posttreatment, defined by the histological detection of high-grade CIN, AIS, or cancer during follow-up, was also evaluated.

### Statistical analysis

Data were entered into an electronic spreadsheet specifically designed for this study, ensuring data confidentiality. Statistical analyses were conducted using SPSS software. Continuous variables are reported using means and SDs. Categorical variables are presented as absolute values and percentages. The correlation between clinical, demographic, and histological characteristics and overtreatment was evaluated by calculating the odds ratio (OR) (Altman, 1991) with a 95% confidence interval. The *χ*^2^ test was used to analyze the association between independent variables in the ‘see-and-treat’ strategy group and the ‘two-step’ strategy group, to make comparisons (Sheskin, 2003). A *P* value less than 0.05 was considered statistically significant.

## Results

During the study period, 2219 patients underwent a cervical excision procedure using LEEP. Of these, 142 patients were excluded from the study because of incomplete clinical, demographic, or histological data. Of the remaining 2077 patients, an additional 895 women were excluded because of initial LSIL, ASC-US, or AGC cytology, or because they underwent the procedure for other reasons, such as symptomatic ectropion or cervical stenosis.

Therefore, in the study period, 1182 patients underwent excision for suspected precancerous lesions with high-grade cytology (ASC-H, HSIL, or suspected cervical cancer). In this group of patients, 275 patients under 25 years of age or pregnant, and 15 patients with negative or CIN1 histological biopsy outcomes were further excluded.

Following the conclusion of the selection process, a total of 892 patients with high-grade cytology were included in the present study and divided into two groups, according to the treatment modality received:

1. ‘See-and-treat’ group: consisting of 643 women who underwent the ‘see-and-treat’ treatment approach;
2. ‘Two-step’ group: consisting of 249 women who underwent the ‘two-step’ treatment approach.

The median age of patients undergoing the ‘see-and-treat’ procedure was 41.5 ± 10.5 years. A total of 119 (18%) patients were menopausal, 189 (29%) patients were smokers, and 32 (5%) patients had immunosuppressive conditions or were taking immunosuppressive medications.

Regarding HPV-related diseases, 33 (5%) patients had a previous diagnosis of cervical, vaginal, or vulvar intraepithelial lesion, and one (0.1%) patient had vulvar cancer, while 46 (7.1%) patients had a concurrent diagnosis of HPV-related disease. Demographic and clinical characteristics of the study population in the ‘see-and-treat’group are reported in Table 1.

**Table 1:** Characteristics of the ‘see and treat’ population

643 Patients	*N* (%) or mean ± SD
Average age	41.5 ± 10.5
Number of patients with previous pregnancies	381 (59.2%)
Number of patients with PV or TC	348 (54.1%)
Number of patients with ABS, IVG, or GEU	80 (12.4%)
Number of patients in menopause	119 (18%)
Number of patients using hormonal therapy	144 (22%)
Number of patients using HRT	15 (2%)
Number of patients using estro-progestins	118 (18%)
Number of patients using IUD	11 (1.7%)
Smoking habit	189 (29%)
Number of patients with HPV-DNA high-risk positive (HPV-DNA hr+)	148 (23%)
Number of patients with HPV-DNA status unknown	492 (76%)
Number of patients with HPV-DNA high-risk negative (HPV-DNA hr−)	3 (0.4%)
Immunosuppression or immunosuppressive therapies	33 (5.1%)
Number of patients with previous HPV-related diseases	65 (10%)
VIN HSIL	3 (0.4%)
Genital warts	31 (5%)
VAIN HSIL	3 (0.4%)
Vulvar or vaginal cancer	1 (0.1%)
CIN HSIL	27 (4.2%)
Concurrent HPV-related diseases	46 (7%)
VIN HSIL	5 (0.7%)
Genital warts	31 (4.8%)
VAIN HSIL	9 (1.3%)
Vulvar or vaginal cancer	1 (0.1%)
HPV vaccination (at least one dose)	64 (9.9%)
HPV vaccination status unknown	465 (72%)
HPV vaccination not done	114 (18%)

ABS, spontaneous abortion; CIN, cervical intraepithelial neoplasia; EP, estro-progestin; GEU, ectopic pregnancy; HPV, human papillomavirus; HRT, hormone replacement therapy; HSIL, high-grade squamous intraepithelial lesion; IVG, voluntary termination of pregnancy; IUD, intrauterine device; PV, vaginal delivery, TC, cesarean section; VAIN, vaginal intraepithelial neoplasia; VIN, vulvar intraepithelial neoplasia.

The baseline cytology results of the ‘see-and-treat’ group are shown in Table 2.

**Table 2:** Baseline cytology results of the ‘see-and-treat’ group

Pap test result	*N* (%)
ASC-H	181 (28.1%)
ASC-H and AGC	3 (0.5%)
HSIL	432 (67.2%)
HSIL and AGC	7 (1%)
Suspected carcinoma	20 (3%)

AGC, atypical glandular cell; ASC-H, atypical squamous cells – cannot exclude high-grade squamous intraepithelial lesion; HSIL, high-grade squamous intraepithelial lesion.

Specifically, 181 (28.1%) patients had ASC-H cytology, 432 (67.2%) patients had HSIL, 10 (1.5%) patients had ASC-H or HSIL with concurrent AGC, and 20 (3%) patients had suspected carcinoma.

The final histological results following cervical excision were: negative outcome in 57 (8.8%) patients, CIN1 in 30 (4.7%) patients, CIN2 in 129 (20%) patients, CIN3/CIS in 365 (57%) patients, microinvasive squamous cell carcinoma in 20 (3.1%) cases, invasive carcinoma in 23 (3.6%) patients, 13 cases of AIS (2%), including eight with concomitant CIN3, five invasive adenocarcinomas (0.7%), and one neuroendocrine tumor (0.15%).

The average width and length of the excised cone were 18.6 ± 8.6 and 16.6 ± 8.4 mm, respectively. The margins were negative in 340 (52.9%) patients and positive in 281 (43.7%) patients, of which 94 were endocervical, 87 ectocervical, and 100 were positive at both the ectocervical and endocervical levels, while in 22 (3.4%) cases they were not assessable. The average time between the colposcopic examination and the excisional treatment was 29 ± 34.9 days. The overtreatment rate, defined as a final histological outcome of CIN1 or negative, was 8.8 and 4.6%, respectively, for a total of 13.4% (87 patients).

A bivariate analysis was performed considering the different anamnestic or clinical factors potentially associated with an increased risk of overtreatment. Specifically, the following factors were considered: menopausal status, ASC-H cytology, nulliparity, lack of hormone therapy, age more than 30 years, a G1 or G0 colposcopic outcome, SCJ type 3, and a positive high-risk HPV DNA test at baseline.

The following factors were found to have the greatest impact on the risk of overtreatment: the grading of colposcopic impression (G1 or G0) and the absence of a visualized SCJ. The respective ORs were 7.36 and 3.57.

The analysis also showed that menopause (OR = 4.5, *P* < 0.0001) and ASC-H cytology (OR = 2.97, *P* < 0.0001) are statistically associated with an increased risk of overtreatment. The exclusion of patients with this cytological result from the cohort reduces the overtreatment rate to 10.6%.

Among the overtreated patients, seven had a positive high-risk HPV test for genotypes 16 or 18 (8%), while 15 were positive for other high-risk HPV genotypes (17%). The analysis indicates that a positive HPV DNA test for genotypes 16 or 18 is associated with a lower risk of overtreatment.

In addition, a subpopulation of 473 patients with a high-grade colposcopic impression (G2+) was selected, thereby identifying a subgroup referred to as ‘high-grade concordance’.

The final histological findings following cervical excision in this population included: negative outcome in 21 (4.4%) patients, CIN1 in nine (1.9%), CIN2 in 89 (18%), CIN3/CIS in 304 (64%), microinvasive carcinoma in 15 (3.1%) cases, AIS in eight (1.7%) cases, including five with concomitant CIN3, invasive adenocarcinoma in five (1%) cases, and invasive carcinoma in 22 (4.6%) cases.

The overtreatment rate in this subgroup of women was 6%. Considering only negative histological outcomes, the overtreatment rate reduces to 4%. In the presence of high-grade cytological-colposcopic concordance, the risk of overtreatment decreases from 13.5 to 6%.

Further analysis indicated that no factors were associated with an elevated risk of overtreatment in the designated ‘high-concordance’ group.

Patients undergoing the conventional ‘two-step’ treatment were finally compared with those in the ‘see-and-treat’ group. The comparison between the two groups is shown in Table 3.

**Table 3:** Comparison of demographic and clinical characteristics between patients in the ‘see-and-treat’ group and in the ‘two-step’ group

Population characteristics	‘See-and-treat’ group (*n* = 643)	‘Two-step’ group (*n* = 249)	*P* value
Average age	41.5 ± 10.5	37.6 ± 8	<0.0001
Previous pregnancies	295 (45.8%)	100 (40%)	0.123 009
Menopause	119 (18%)	18 (7%)	0.000 028
Smoking	189 (29%)	86 (34.5%)	0.502 426
Hormone therapy	144 (22%)	57 (22.8%)	0.149 059
Previous or concomitant HPV-related pathologies	83 (12.9%)	31 (12.4%)	0.85 404

HPV, human papillomavirus.

Statistically significant differences in patient age and menopausal status were found between the two groups.

The final histological outcomes following cervical LEEP procedures included: negative outcome in two (0.8%) patients, CIN1 in 12 (4.8%) patients, CIN2 in 46 (18.4%) patients, CIN3/CIS in 178 (71.4%) patients, microinvasive carcinoma in three (1.2%) cases, one case of AIS (0.4%), one invasive adenocarcinoma (0.4%), one microinvasive adenocarcinoma (0.4%), and invasive carcinoma in five (2%) patients.

In the ‘two-step’ approach group, the risk of biopsy underdiagnosis was calculated, defined as the detection of invasive carcinoma after treatment, following a biopsy that was not indicative of invasion. Regarding the definitive cancer diagnosis, 10 invasive tumors were identified, seven of which were identified after excisional treatment (2.8%). In addition, out of 80 patients with a biopsy result of CIN2, 49 patients had a definitive histological outcome of CIN3 (61%). Of these patients, six (12%) were under 30 years of age. In this group, the overtreatment rate of excisional treatment after a biopsy histological outcome of CIN2+ was calculated, resulting in 5.6%.

Moreover, the time interval between colposcopy and LEEP, the status of excised cone margins, the size of the cone, any recurrences of CIN, AIS, or cancer within 3 years, and the number of patients lost to follow-up were compared between the two groups. The results of these comparisons are presented in Table 4.

**Table 4:** Clinical characteristics after treatment between the two study groups

Clinical characteristics	ʻSee-and-treat groupʼ (*n* = 643)	‘Two-step group’ (*n* = 249)	*P* value	*χ* ^2^
Time interval between colposcopy and LEEP	29 days ± 34.9	68.8 days ± 53	<0.0001	
Presence of positive margins	281 (43.7%)	99 (39.7%)	<0.0001	23.409
Cone length (mm)	16.6 ± 8.4	16.6 ± 5.6	1	
Cone width/mm)	18.6 ± 8.6	19.5 ± 4.6	0.1176	
Recurrences	69 (10.7%)	17 (6.8%)	0.0744	3.183
Patients lost at follow-up	203 (31.5%)	80 (32%)	0.872 443	0.0258

LEEP, loop electrosurgical excision procedure.

The *χ*^2^ calculation showed statistically significant differences between the two groups with regard to the time elapsed between colposcopy and intervention (29 days in the ‘see-and-treat’ group vs. 68 days in the ‘two-step’ group), as expected, and margin positivity.

## Discussion

Following the cytological finding of high-grade cervical abnormalities, the subsequent diagnostic-therapeutic process involves a second-level colposcopy examination.

At this stage, the colposcopist may confirm the initial cytological suspicion or, conversely, have a discordant impression regarding the likelihood of a high-grade cervical lesion. When cytology and colposcopy are discordant, or when the SCJ is not fully visible, biopsy or endocervical sampling are recommended to guide further management. Conization becomes mandatory in women with persistent HSIL cytology at follow-up, or is recommended in cases of ASC-H or HSIL cytology with a nonvisible or partially visible SCJ, to obtain histological confirmation on a representative tissue sample.

Several authors propose the ‘see-and-treat’ approach, which aims to optimize both the diagnostic phase with colposcopy and the therapeutic phase of cervical excision, without performing an intermediate biopsy. The risk of such treatment may be a higher overtreatment rate compared with the standard ‘two-step’ approach because it relies on the subjective impression of the colposcopist who may be influenced by the initial cytological finding and may be more likely to identify high-grade cervical abnormalities (Kietpeerakool *et al*., 2007; Bosgraaf *et al*, 2013; Ebisch *et al*., 2016; Kuroki *et al*., 2016; Ciavattini *et al*., 2019; Loopik *et al*., 2020; Rongthongaram *et al*., 2023).

The present study aimed to evaluate the overtreatment rate in patients managed with the ‘see-and-treat’ approach. In our case series, the overtreatment risk was found to be 13.5%. When considering only patients with high concordance between high-grade cytology and colposcopic impression G2 or higher, the overtreatment percentage reduces to 6%. Furthermore, in this high-risk population where histological outcomes following excision were negative for CIN (no evidence of CIN), the overall prevalence of overtreatment was 4%. This data is significant, considering that for high-grade cytology with histological confirmation of CIN1, an excisional diagnostic procedure is considered acceptable (SICPCV Gestione delle lesioni istologiche, 2019; Perkins *et al*., 2020).

Our results show how the risk of overtreatment can be minimized if the ‘see-and-treat’ approach is undertaken following high-grade cytology and colposcopic impression, emphasizing the decisive role of colposcopy.

Several studies in the literature examine the ‘see-and-treat’ strategy, comprising heterogeneous groups of patients, both in terms of age at the time of treatment, Pap test results, criteria established for performing a ‘see-and-treat’, and the different excision techniques used. This variability has led to heterogeneous rates of overtreatment being reported (Kietpeerakool *et al*., 2007; Aue-Aungkul *et al*., 2011; Bosgraaf *et al*, 2013; Guducu *et al*., 2013; Kim *et al*., 2014; Blain *et al*., 2016; Ebisch *et al*., 2016; Kuroki *et al*., 2016; Nghiem *et al*., 2016; Smith *et al*, 2016; Waxman, 2016; Ciavattini *et al*., 2019; Loopik *et al*., 2020; Bonow *et al*, 2022; Kiviharju *et al*., 2022; Sachan *et al*., 2022; Rongthongaram *et al*., 2023).

Some of the studies reported in the literature are consistent with our results (Table 5).

**Table 5:** Comparison with studies on ‘see-and-treat’ approach

Authors	Study type	No. of cases	Population type	OT (CIN1 or negative) with high-grade cytology
Loopik *et al*. (2020)	Retrospective	36 581	Low- and high-grade cytology; colposcopy grade not specified	• 11.3%
Ciavattini *et al*. (2019)	Retrospective	254	Age more than 25 years; high-grade cytology; visible SCJ	• 12.6% • High-grade concordance: 3.2%
Kietpeerakool *et al*. (2007)	Retrospective	446	High-grade cytology (HSIL); colposcopy grade not specified	• 5.8% (OT if negative outcome)
Rongthongaram *et al*. (2023)	Retrospective	220	High-grade cytology; colposcopy G0, G1, and G2	•11.4%
Bosgraaf *et al*. (2013)	Retrospective	3192	Low- and high-grade cytology; colposcopy G1 e G2	•18.1% • High-grade concordance: 4.5%

CIN1, cervical intraepithelial neoplasia; OT, overtreatment; HSIL, high-grade squamous intraepithelial lesion; SCJ, squamocolumnar junction.

Similarly, in the study conducted by Bosgraaf and colleagues, the overall overtreatment rate among women managed with the ‘see-and-treat’ approach was 18.1 and 4.5% among women with a high-grade concordance between cytological and colposcopic findings (Bosgraaf *et al*, 2013).

The systematic review conducted by Ebisch et al. (2016), published in BJOG in 2015, estimated the overtreatment rates of ‘see-and-treat’ approach in women undergoing treatment for suspected CIN, in relation to the reference Pap test and colposcopic impression. Thirteen studies were examined between 1996 and 2014, including a total of 4611 patients. The overtreatment rate in women with high-grade Pap test and low-grade colposcopic impression was 29.3%, while in the case of low-grade cytology and high-grade colposcopic impression, it was 46.4%. An overtreatment rate of 11% was found in cases of immediate excisional treatment after high-grade cytology and colposcopic impression (high-grade concordance). This rate is higher than that obtained in our study (6%), which is lower than the 10% threshold defined as acceptable by the British Society and Cervical Pathology Guidelines (Luesley and Leeson, 2010).

A recent review by Loopik et al. (2020) included 36 581 women, of which 10 713 underwent a two-step approach, and 6851 underwent a ‘see-and-treat’ approach. This review aimed to compare the overtreatment rate in the ‘see-and-treat’ and in the ‘two-step’ approaches, and evaluate the best approach to apply to clinical practice in the Netherlands. The mean time between cytology and treatment was found to be shorter in women who received the ‘see-and-treat’ approach (1 month) compared with the ‘two-step’ approach (2 months), as also evidenced by the data presented in this study (29 vs. 68 days, respectively) (*P* < 0.0001). In the study previously mentioned, of 4677 undergoing the ‘see-and-treat’ approach with high-grade cytology, a total of 529 women (11.3%) were overtreated. It also emerged that women over the age of 40 and women who were HPV-negative were more likely to be overtreated. An important limitation of the study conducted by Loopik *et al*., was the omission of considering the colposcopic impression.

In the bivariate analysis of our study, the colposcopic impression was found to be essential in determining the treatment choice and in reducing the risk of overtreatment. It can be posited that this data is indicative of the expertise of the colposcopic operators (in our center, colposcopists have a minimum of 10 years of colposcopic experience) and emphasizes the role of subjective clinical experience in colposcopy (Qin *et al*., 2023).

A recent multicenter retrospective Italian study by Ciavattini *et al*. (2019) found low overtreatment rates, both in the general population (12.6%) and in the high-grade concordance population (3%). The study involved a total of 254 patients with an average age of 38 years, including women aged 26 years and over. The analysis excluded colposcopy procedures where the SCJ was not visible. Conversely, in our study, cases in which the SCJ was not visible during colposcopy were considered, as colposcopy is considered adequate even if the junction itself is not visible (Bornstein *et al*., 2012); however, the bivariate analysis conducted in our case series on the association between clinical-anamnestic factors and the risk of overtreatment highlighted that a SCJ type 3 significantly increased the probability of overtreatment (OR = 3.5730, *P* < 0.0001). This data is not confirmed in the high-grade concordance group, where the colposcopic impression G2 is a determining factor, regardless of the visibility of the SCJ. Among the factors considered in the study by Ciavattini *et al*., (age, menopause, and colposcopy grade 1), a positive association was found only between overtreatment and colposcopy grade 1, while age and menopause did not influence the overtreatment rate. Our study suggested instead that, in addition to the colposcopic impression, other factors may increase the risk of overtreatment, including menopausal status, ASC-H cytology, and a positive high-risk DNA test other than 16 or 18.

Similarly, in a retrospective study by Kietpeerakool et al. (2007), menopause emerged as a significant independent risk factor, associated with a threefold increase in the likelihood of receiving excessive treatment after ‘see-and-treat’ excision compared with women of childbearing age.

The importance of HPV DNA evaluation for the management of patients undergoing the ‘see-and-treat’ approach was also emphasized by Rongthongaram *et al*., particularly within the group with ASC-H cytology, where the overtreatment rate reported in the literature is quite heterogeneous (Guducu *et al*., 2013; Sachan *et al*., 2022; Rongthongaram *et al*., 2023). In this retrospective study, 220 patients were treated, with an overtreatment rate of 11.4%. In patients with ASC-H cervical cytology, the overtreatment rate increased 13 times. According to the ASCCP 2019 risk estimation supporting the consensus guidelines, the risk of immediate CIN3+ in women with ASC-H was 3.4% for those who were HPV negative, and 26% for those who were HPV positive. Considering the high risk of immediate CIN3+ in HPV-positive women with ASC-H, a ‘see-and-treat’ approach would be appropriate; however, in HPV-negative women with ASC-H, a two-step approach should be more relevant, especially in those with fertility concerns.

Therefore, women with ASC-H cervical cytology should be managed more conservatively based on HPV test results (Rongthongaram *et al*., 2023).

Our data showed that, excluding patients with ASC-H cytology, the overtreatment rate was reduced to 10.6. Other studies, however, do not confirm this finding (Guducu *et al*., 2013; Sachan *et al*., 2022).

An additional evaluation conducted in the ‘two-step’ group was the analysis of the risk of biopsy underdiagnosis, that is, estimating the cases where a nonsuggestive biopsy outcome for tumor pathology results in a definitive histology of invasive lesion, which was found to be 2.8%. Potentially, a ‘see-and-treat’ approach could reduce the duration of the diagnosis and treatment for invasive lesions that remained occult to biopsy. In women with poor adherence to therapy and follow-up, the ‘see-and-treat’ approach might be appropriate to minimize the risk of unrecognized occult invasive lesions, as suggested in other studies (Aue-Aungkul *et al*., 2011; Guducu *et al*., 2013; Ciavattini *et al*., 2019).

Finally, in the present study, it was observed that 12% of patients who underwent a biopsy with a CIN2 outcome and a subsequent definitive diagnosis of CIN3 after treatment were under 30 years of age. Literature suggests that CIN2 lesions in women under 30 years of age have a higher regression rate and may not require treatment, thus necessitating a more conservative approach (Castle *et al*., 2009; Silver *et al*., 2018; Tainio *et al*., 2018; Kiviharju *et al*., 2022; Lycke *et al*., 2024). Our patients under 30 years of age with a histological outcome of CIN2 could have been conservatively treated based only on the biopsy result; however, the study results suggest that these women could have benefited from the ‘see-and-treat’ approach, which would have avoided the loss of occult CIN3 lesions in 61% of cases.

### Limitations and strengths

The main limitation of the present study is its retrospective nature, which entails the collection of data from existing medical records. Such a methodological approach is subject to a high rate of unreported information and the potential for errors.

The main strengths are twofold. First, colposcopies were performed by a limited number of operators adequately trained in the diagnosis and treatment of HPV-related pathologies, ensuring standardized results and reducing interindividual variability. Second, the large number of recruited patients enabled the acquisition of a representative sample, thus yielding statistically significant results.

### Conclusion

The ‘see-and-treat’ approach appears to be a valid alternative for the management of preneoplastic lesions of the uterine cervix. The most important strengths of this strategy include a shorter interval from diagnosis to treatment, lower costs, and a reduction in patients' anxiety while waiting for the final histological result; however, the main advantage lies in the possibility of early diagnosis and treatment of pretumoral and invasive lesions that may remain occult to biopsy.

The issue of underdiagnosed invasive neoplasm detected in our case series is particularly relevant, and compensates the risk of overtreatment. This is especially true in settings that, unlike ours, are not referral centers.

Furthermore, by reducing the number of visits and necessary controls, it ensures greater patient adherence to therapy, as well as cost reduction; however, it is crucial to accurately identify patients eligible for the ‘see-and-treat’ approach to limit the rate of overtreatment, as in cases where colposcopy does not confirm the suspicion of high-grade cytology, given that the risk of overtreatment (13.5%) is not particularly advantageous. The ‘see-and-treat’ approach may be more advantageous in cases with initial high-grade cytology and G2+ colposcopic impression (where there is a high degree of concordance), and in cases where there are no factors increasing the risk of overtreatment, considering that an estimated risk of 6% is considered acceptable (Luesley and Leeson, 2010).

## Acknowledgements

This study was partially supported by the Italian Ministry of Health – Current Research IRCCS.

Conceptualization, methodology, data curation, writing – original draft: E.M. Data curation, writing – original draft: C.M. Conceptualization, statistical analysis, data curation, writing – original draft: C.M.M.M. Data collection, visualization: M.S. and M.P.C. Conceptualization and methodology: E.D.L., G.L., V.B., and D.A. Supervision, project administration, writing – review and final approval: G.B.

### Conflicts of interest

There are no conflicts of interest.
